# Experimentelle und Kritische Beiträge zur Händedesinfectionsfrage

**Published:** 1902-09

**Authors:** 


**Experimentelle und Kritische Beiträge zur Händedesinfectionsfrage.** Von Dr. Richard Schaeffer. Pp. 110. Berlin: S. Karger. 1902.

No more important questions can engage the attention of surgeons and obstetricians than those relative to the disinfection of the hands. The book before us is a notable contribution to the literature of the subject, and deserves most attentive study.

It may be taken as a truism that there are no means by which the hands can be rendered absolutely germ-free. The author's object in this work has been to ascertain to what degree and by what means the ideal condition of absolute sterility of the hands can be most nearly reached in practice. He records the numerous bacteriological investigations he has made in the attempt to solve the problem, and these investigations appear to have been carried through with every precaution against error that ingenuity could suggest. In addition the work of others in the same field of research is freely criticised.

As a result of his labours, the author declares that the hot water and alcohol method of disinfection of the hands of Ahlfeld gives by far the best results. It is time, he says, to give up the idea that any antiseptic solution as ordinarily used can be relied upon to effectually disinfect the hands. The value of alcohol depends not upon its bactericidal power, but upon its power of cleansing the skin and removing fat and epithelium. After cleansing in alcohol the hands should be washed in sterile fluid to remove the alcohol and the germs it has extracted.

In greater detail the method of disinfection advocated by the author consists: Firstly, in washing the hands for five minutes in very hot water (50° C.), using for the purpose soft soap and a sterile brush and changing the water several times; secondly, in rubbing the hands vigorously with a sterile towel; thirdly, in brushing the hands in alcohol for from three to five minutes and changing the alcohol at least once during the process; fourthly and finally, in rinsing the hands in 1 in 1000 perchloride of mercury solution. Next in merit to the method described is the soap and spirit method of Mikulicz.

